# Carbohydrate Intake and Closed-Loop Insulin Delivery System during Two Subsequent Pregnancies in Type 1 Diabetes

**DOI:** 10.3390/metabo12111137

**Published:** 2022-11-18

**Authors:** Ana Munda, Chiara Kovacic, Drazenka Pongrac Barlovic

**Affiliations:** 1 Department of Endocrinology, Diabetes and Metabolic Diseases, University Medical Centre Ljubljana, 1000 Ljubljana, Slovenia; 2 Medical Faculty, University of Ljubljana, 1000 Ljubljana, Slovenia

**Keywords:** algorithm-controlled insulin delivery systems, real-time continuous glucose monitoring, closed-loop insulin pump, carbohydrate intake, meal frequency, pregnancy

## Abstract

Carbohydrate intake is one of the main determinants of glycemic control. In pregnancy, achievement of tight glycemic control is of utmost importance; however, data on the role of hybrid closed-loop systems (HCLs) in pregnancy are scarce. Therefore, we aimed to assess glycemic control achieved through the use of HCLs, and its association with carbohydrate intake in type 1 diabetes pregnancy. We included data from women with a sensor-augmented pump (SAP) during their first pregnancy and HCL use during the subsequent pregnancy. Student’s paired t-test was used to compare data between both pregnancies. Six women were identified, with age 30.2 ± 3.6 vs. 33.0 ± 3.6 years, diabetes duration 23 ± 5 vs. 26 ± 5 years, and baseline HbA_1c_ 6.7 ± 0.7% (50.1 ± 7.7 mmol/mol) vs. 6.3 ± 0.6% (45.2 ± 6.5 mmol/moll) in the first and second pregnancies, respectively. Time with glucose in the range 3.5–7.8 mmol/L was 69.1 ± 6.7 vs. 78.6 ± 7.4%, *p* = 0.045, with the HCLs compared to SAP. Higher meal frequency, but not the amount of carbohydrate consumption, was associated with more time spent in the target range and lower glycemic variability. HCLs and meal frequency were associated with better glycemic control in a small series of pregnant women with type 1 diabetes. Whether this translates to better perinatal outcomes remains to be seen.

## 1. Introduction

Pregnancy is a challenging life period, characterized by dynamic changes in insulin sensitivity and glucose tolerance, and demands the achievement of tight glycemic control [1]. Carbohydrate intake is one of the main determinants of glucose control. Guidelines suggest that the minimal daily carbohydrate intake should be 175 g [2]. However, the optimal carbohydrate intake during type 1 diabetes pregnancy was not tested in interventional trials and remains ill-defined.

Recently, continuous glucose monitoring (CGM) systems, with and without insulin pump therapy, have revolutionized diabetes care [3]. In particular, new algorithm-controlled insulin delivery systems based on real-time CGM, also named hybrid closed-loop systems (HCLs), have changed the clinical landscape, by providing new therapeutic targets, as well as an increase in the proportion of people with type 1 diabetes safely achieving those goals [4]. However, the role of these systems in pregnant women with type 1 diabetes is still not well characterized.

Therefore, we aimed to assess glycemic control in women with type 1 diabetes during pregnancy and using HCLs, and to compare it to the glycemic control in their previous pregnancy, when they used a sensor-augmented pump (SAP). In particular, we were interested in how achievement of the correct level of glycemic control was associated with the average daily carbohydrate intake and the number of meals. In addition, we wanted to analyze user experience with the HCLs and contrast it to the experience with the use of SAP during pregnancy.

## 2. Materials and Methods

This study utilized clinical data abstracted from medical records of multiparous women with type 1 diabetes who received care at the Department of Endocrinology, Diabetes and Metabolic Diseases at the University Medical Center Ljubljana. We included women who had used an insulin pump with an advanced hybrid closed-loop algorithm, HCLs (Minimed 780G, Medtronic, Northhridge, CA, USA), in their most recent pregnancy (until the end of September 2022). In addition, to participate in this analysis, women must have had experience with an SAP (Minimed Paradigm Veo or Minimed 640G system with a matching CGM sensor, all Medtronic, Northhridge, CA, USA) during their previous pregnancy. 

Women eligible for this study were 18 to 40 years old, with a singleton fetus and with glycated hemoglobin (HbA_1c_) 6.0 to 10.0% (42 to 86 mmol/mol). All women gave informed consent to treatment with the HCLs during pregnancy. The Minimed 780 g pump system uses an insulin pump, CGM data, and a control algorithm to adjust the amount of insulin infused in real-time, with a new autocorrection bolus. All women set the target glucose rate at 5.5 mmol/L (the lowest of the pre-set options). They retained the ability to make the system more or less aggressive through adjustment of the insulin settings. Since this is a hybrid closed-loop system, manual meal boluses of insulin are delivered based on carbohydrate counting. All women attended an update in education regarding carbohydrate counting and self-management of diabetes at the beginning of the first and the second pregnancy. In addition, they all received education regarding the technical aspects of insulin pump use and CGM devices.

The CGM and insulin pump data were analyzed for every trimester of the most recent pregnancy and compared to the previous pregnancy’s glycemic data. In addition, the women were asked to complete a questionnaire, designed with the aim of better understanding their satisfaction with the advanced hybrid closed-loop algorithm during pregnancy. The questionnaire had 10 open-ended and eight closed questions. Open-ended questions assessed satisfaction with the use of the HCLs, its advantages and disadvantages, the experience of handling the HCLs when entering the carbohydrate amount, and suggestions for improvements. Closed questions were used to assess the frequency (from 1-twice per week or less to 55-multiple times per day), intensity (1-low, 2-moderate, 3-high), and time (1–30 min or less, 2-between 30 and 60 min, 3–60 min or more) of physical activity, the occurrence of nausea and vomiting, and the perception of the hypoglycemia occurrence (3 categories: the same/better/worse than in the previous pregnancy).

All women received specialist antenatal care from a multidisciplinary team, including gynecologists, endocrinologists, nurse educators, ophthalmologists, dieticians, and psychologists. Clinic visits were scheduled every 2 to 4 weeks. In addition, their glycemic data were reviewed weekly, through online communication. Participants’ weight, blood pressure, insulin dose, adverse events, and episodes of severe hypoglycemia (defined as an event requiring third-party assistance) were recorded at each visit, and HbA_1c_ was measured. 

Data on concomitant diseases and chronic diabetes complications were obtained from medical records at the first prenatal visit. Diabetic nephropathy was defined based on macroalbuminuria, with a urinary albumin-creatinine ratio ≥300 mg/g [5] within the year prior to the first prenatal visit. 

The study was conducted in accordance with the guidelines of the Declaration of Helsinki and Good Clinical Practice. It was approved by the Slovenian Ethics Committee.

### Statistical Analyses

CGM data were analyzed from the raw glucose data for each trimester separately, i.e., before the 12th, the 24th, and 34th gestational weeks and compared in the same women in both pregnancies. We compared the mean CGM glucose concentration, coefficient of variation, glucose management indicator (GMI), and percentage of time spent in the target glucose range. The target glucose range was defined as a glucose concentration of 3.5–7.8 mmol/L (63–140 mg/dL), and time spent in this range (TIR), together with the time spent below a glucose concentration of 3.5 mmol/L (63 mg/dL) (TBR) and the time spent above a glucose concentration of 7.8 mmol/L (140 mg/dL) (TAR), was compared between both pregnancies. GMI is a metric that approximates the expected laboratory HbA_1c_ level based on average glucose measured using CGM values [6]. In addition, we recorded data on the carbohydrate intake and insulin dose. Specifically, we were interested in how the HCL algorithm ensured insulin delivery for carbohydrate intake. Therefore, we described the associations between carbohydrate intake and the number of meals with glycemic control using Pearson’s correlation coefficients.

Data analysis was carried out using SPSS Statistics version 21 (IBM, Armonk, NY, USA) and the software R (R Foundation for Statistical Computing, Vienna, Austria (package CGManalyzer). The normally distributed descriptive statistics of continuous data were presented as means, with the corresponding standard deviations. Variables that deviated from the normal distribution were presented as medians with interquartile ranks. Student’s paired *t*-test was used to compare glycemic data between both pregnancies for normally distributed data. In the case of deviations from the normal distribution, a Wilcoxon signed-rank test was used. A 5% significance level was used for all comparisons, without adjustment for multiplicity. A post hoc power analysis was conducted using G*power 3.1.7 [7].

A qualitative content analysis [8] was used to analyze open-ended questions, to better understand the women’s experience when using HCL. Using this technique, extensive texts were classified into smaller content categories. Data preparation was followed by data organization, including open coding, grouping the codes, and abstraction. The coding process was performed by AM and DBP. First, they independently coded all texts. Later, they tried to achieve an intercoder agreement. The sample was convenient, therefore we did not aim for data saturation.

## 3. Results

Up to the end of September 2022, we identified six women with type 1 diabetes treated with a SAP in one pregnancy and with the HCLs during the subsequent pregnancy. Three of those women started using the HCLs before the second pregnancy, whereas three women switched to the HCLs at the beginning of the second pregnancy. The clinical characteristics of the study population are presented in Table 1. There were, on average, almost 3 years between the first and second pregnancy. Women started the second pregnancy with a numerically lower HbA_1c_; however, the difference was not statistically significant. None of the study participants experienced severe hypoglycemia or acute hyperglycemic complications during the pregnancies. After, on average, more than 20 years of type 1 diabetes duration, two-thirds of women had incipient diabetic retinopathy and five out of six had impaired hypoglycemia awareness. No other late diabetes complications were identified.

Although there was no significant difference in the mean sensor glucose concentration, HbA_1c_, or GMI found between the HCLs and SAP across pregnancy trimesters, we found some differences in glycemic control (Table 2). TIR was on average higher by 9.5% in the second trimester when using the HCLs (achieved statistical power was 0.71). It increased further in the third trimester, to almost 84%. With the HCLs, less time was spent with glucose above 7.8 mmol/L (16.6 vs. 26.6% in the second trimester) with 0.72 achieving statistical power and less time also in the hypoglycemic range (2.6 vs. 5.9%), although the latter was not statistically significant. Moreover, the coefficient of variation was significantly lower in the third trimester when using the HCLs. Of note, the total daily insulin dose did not differ between the two systems, even though the HCLs delivered more bolus insulin and less basal insulin per body weight than the SAP system (Table 2). The achieved statistical power for bolus and basal insulin ranged from 0.76 for the percentage of daily bolus and basal insulin, to 0.99 for total basal insulin. In addition, the achieved power for the coefficient of variation in the 3rd trimester was 0.99.

A higher meal frequency with the HCLs was associated with a higher TIR and less time spent with a glucose concentration above 7.8 mmol/L. Furthermore, higher meal frequency was associated with a lower coefficient of glucose variability (Table 3). There were fewer automatic correction boluses needed when the number of meals was higher (Table 3). However, the amount of carbohydrates was not significantly associated with any of the glycemic parameters (Table 3). With the SAP, there was no association found between the meal frequency or amount of carbohydrate intake and the parameters of glycemic control (Supplementary Table S1).

**Figure 1:**
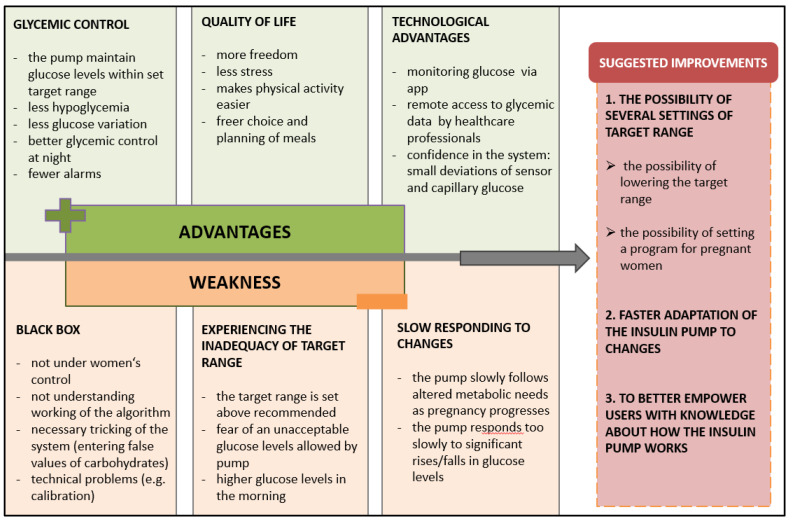
Results of the qualitative content analysis.

The results of the qualitative content analysis are presented in Figure 1. Three main categories were formed: (1) advantages of using the HCLs, (2) weaknesses of using the HCLs, and (3) suggested improvements. Overall, the women’s satisfaction with the HCLs was high. The main advantage of the HCLs identified was an easier achievement of the target glucose range. In addition, a lower number of hypoglycaemias, especially at night, contributed to an improved quality of life, with less stress and more freedom in everyday choices. Some participants emphasized fewer dietary restrictions when using the HCLs. In addition, engaging in physical activity was easier; however, reported physical activity intensity, frequency, and duration did not differ between both pregnancies (Supplementary Table S2). They recognized the advantage of modern technologies in easier glucose concentration monitoring via apps that also enable remote consultations with healthcare professionals. On the other hand, all of the participants pointed out that the main disadvantage of the HCLs was the feeling of not having full control over the pump and not always understanding its algorithm. They were also not satisfied with the slow response of the HCLs to increases in glucose concentration. Moreover, they could not choose a target glucose lower than 5.5 mmol/L, which many times caused them to fear that they would not be able to achieve good pregnancy outcomes, especially at the beginning of pregnancy. Regardless, all studied women would choose to use the HCLs again in their next pregnancy. At the same time, they suggested some improvements related to the pump settings (Figure 1).

## 4. Discussion

In the present study, we have shown that, when using the commercially available HCLs Minimed 780G, women spent more time in the target glucose range, less time above the target range, and had lower glucose variability compared to their previous pregnancy, when they used a SAP. In addition, we have demonstrated that with the HCLs, the amount of carbohydrate consumption was not associated with glycemic parameters. However, higher meal frequency was associated with more time spent in the glucose target range, less time spent in hypoglycemia, and with lower glycemic variability (Table 4).

Very limited data exist about the use of closed-loop insulin delivery systems during type 1 diabetes pregnancy. In a short-term randomized controlled study of 16 women using a closed-loop system, the DANA Diabecare R Insulin Pump and the FreeStyle Navigator II, they reported higher TIR and lower average glucose concentrations during 4 weeks of overnight use [9]. However, during day-and-night usage of the same system in 16 pregnant women, the proportion of time with glucose levels within the target, as well as mean glucose concentration, were comparable during closed-loop and SAP insulin delivery [9]. However, with the closed-loop, fewer hypoglycemic episodes occurred [10]. We found also three case reports of the use of a closed-loop system during pregnancy, using DexCom G6 sensors, an online open-source platform Nightscout, and a pump with two-way communication capabilities, and achieving favorable glycemic control; however, they reported a few technical difficulties [11,12]. There is also a report on a case series available, from three women using a Minimed 670G hybrid closed-loop system; however, that system has a target glucose concentration set at 6.7 mmol/L, which is considerably higher than the recommended target range in pregnancy [13]. 

In the current study, we described for the first time, to the best of our knowledge, the glycemic outcomes of pregnant women using the commercially available HCLs Minimed 780G, followed through pregnancy as part of our standard clinical care. Across pregnancy, the ambition is to increase the TIR, while reducing TAR, TBR, and glycemic variability measures. In the second trimester, in our study, the percentage of TIR while using HCLs was almost 10% higher compared to the previous pregnancy, with the use of SAP. Moreover, women reached, on average, 83.6% TIR in the last trimester when using the HCLs. According to the published data, pregnant women with type 1 diabetes currently spend, on average, 50%, 55%, and 60% in the TIR during the first, second, and third trimesters, respectively [14,15]. TIR increased to 68% in the 3rd trimester in the well-known randomized controlled trial CONCEPTT [16], which was still below the recommended >70% [3]. 

Our data show that the recommended targets can be achieved to a greater extent with the HCLs. What is more, they can be achieved from the first trimester onwards. This is crucial, especially in light of new analyses of CGM data during pregnancy, showing that, for adverse neonatal outcomes, early pregnancy glycemic metrics are even more important than previously thought [17].

We have demonstrated that a higher meal frequency, but not a greater amount of carbohydrate intake, was associated with more time spent in the target range, less time spent in hypoglycemia, and with lower glycemic variability. The positive association of meal frequency with better glycemic control was also shown with some insulin pump systems in other studies [18]. Possibly, a higher number of meals and, thus, more insulin boluses being delivered enable a better performance of the automatic algorithm, with a more successful prevention of glucose increases above the target range. It remains to be seen whether women on a low-carbohydrate diet, possibly with fewer insulin boluses per day, can achieve a similar glycemic control using the HCLs compared to the SAP.

Importantly, women did not report any serious adverse events with the HCLs or SAP system. They expressed overall great satisfaction with the HCLs, especially because of having less hypoglycemia and more freedom in their everyday diet and physical activity plans. However, they disliked the somehow slow response to glucose increase correction by the commercially available HCLs. This is most probably because this system was not designed primarily for use in pregnancy, and its main goal is safety, with a primary focus on effective hypoglycemia prevention. For the pregnancy outcomes this might not be optimal, since a detailed analysis of >10.5 million CGM glucose measures from two large multicenter pregnancy trials showed that normal offspring birth weight was associated with a TBR well above the recommended international consensus target of ≤4%, with the TBR never falling below 8% in women with normal-sized babies [17]. 

Our study is the first report of glycemic outcomes with the commercially available HCLs Minimed 780G in pregnancy. In addition, we compared glycemic outcomes to the ones from a previous pregnancy, when using a SAP system, in the same sample of women. The best way to compare the two systems would be a large multicentric randomized controlled trial, which is currently underway. However, since glucose control in pregnancy is very demanding and women struggle to achieve tightly set targets, these reports may be helpful in everyday clinical practice. In addition, we included personal experience with the HCLs, as reported by the pregnant women. This enabled us to understand that, e.g., although we found no role in the amount of ingested carbohydrates with glycemic parameters, women reported more flexibility in their food choices, while retaining good glycemic control with the HCL system. 

The main limitation of our study is the small sample size. However, we tried to at least partly overcome this limitation by exploring glucose control in the same sample of women, in this way eliminating or decreasing the effect of many psychosocial factors, which are crucially important for good glycemic control during pregnancy, i.e., education, understanding, dedication, the level of physical fitness, and the manner of eating. In the second pregnancy, these women already had experience of managing glucose during pregnancy; however, they had less time for dedicated diabetes management, since they needed to take care of the small child they already had. Another limitation of this study is the lack of reported perinatal outcomes. Namely, half of the women included had not delivered at the end of September 2022, and thus we could not analyze perinatal outcomes in both pregnancies; also, the sample is small for such comparisons. Nevertheless, analysis of perinatal outcomes would be very important, since a recent large cohort study of type 1 diabetes pregnancies from the Joslin diabetes center pointed out that, although CGM use was associated with better glycemic control (reflected by lower HbA_1c_), it did not translate into a significant improvement of any of the maternal or neonatal outcomes [19]. We also lack detailed information on diet and the way the meals were structured, since our analysis only included the amount of ingested carbohydrates. The amount and the quality of proteins and fats in the meal may be as important as carbohydrates for the glycemic and pregnancy outcomes; therefore, it would be necessary to have those data for a detailed understanding of pregnancy outcomes, with regard to glycemic control in type 1 diabetes.

To conclude, in the present study we have shown that using the new technologies for insulin delivery may be beneficial in achieving target glucose control and alleviating the diabetes burden during pregnancy. In addition, we have demonstrated that the amount of carbohydrates consumed was not associated with glycemic control in our sample of women. More research needs to be done to characterize women who would gain the most benefits from the new HCLs system, also based on meal frequency and meal patterns. Above all, how this translates to improved perinatal outcomes remains to be seen.

## Figures and Tables

**Table 1:** Clinical parameters of the pregnant women studied.

	First Pregnancy	Second Pregnancy	*p*-Value
Age (years)	30.2 ± 3.6	33.0 ± 3.6	<0.001
Diabetes duration (years)	23 ± 5	26 ± 5	<0.001
Education (bachelor’s degree or higher), n (%)	6 (100%)	6 (100%)	NA
Smoking, n (%)	0	0	NA
Pre-conception BMI (kg/m^2^)	23.1 ± 3.1	23.7 ± 4.1	0.347
Pre-conception HbA_1c_, %	6.7 ± 0.7	6.3 ± 0.6	0.100
mmol/mol	50.1 ± 7.7	45.2 ± 6.5	
GWG (kg)	12.0 ± 4.0	9.2 ± 4.9	0.433
SBP, 1st trimester (mmHg)	119.5 ± 11.5	125.0 ± 5.4	0.122
DBP, 1st trimester (mmHg)	71.0 ± 13.9	72.5 ± 13.6	0.707
Nausea during pregnancy, n (%)	4 (66.7)	5 (83.3)	0.317
Vomiting during pregnancy, n (%)	1 (16.7)	1 (16.7)	NA
Concomitant disease	Number of participants		
Celiac disease	1		
Juvenile arthritis	1		
Hashimoto thyroiditis Retinopathy, non-proliferative	1 4		
Retinopathy, pre-proliferative/proliferative	0		
Diabetic kidney disease	0		
Impaired hypoglycemia awareness	5		

BMI—body mass index, GWG—gestational weight gain, SBP—systolic blood pressure, DBP—diastolic blood pressure. Data are presented as mean ± SD or numerous (%).

**Table 2:** Comparison of glycemic parameters between the first and the second pregnancies.

	First Trimester			Second Trimester			Third Trimester		
	First Pregnancy	Second Pregnancy	*p*-Value	First Pregnancy	Second Pregnancy	*p*-Value	First Pregnancy	Second Pregnancy	*p*-Value
Mean SG, mmol/L	6.6 ± 0.5 (6.1, 7.3)	6.4 ± 0.1 (6.3; 6.4)	0.581	6.6 ± 0.3 (6.4, 7.0)	6.1 ± 0.4 (5.7, 6.7)	0.067	5.8 [5.7–6.0] (5.7, 6.7)	6.4 [6.2–6.4] (5.6, 6.4)	0.345
TIR, %	71.7 ± 6.2 (65.8, 80.0)	73.7 ± 9.5 (65.8, 80.0)	0.701	69.1 ± 6.7 (61.5, 76.0)	78.6 ± 7.4 (69.0, 89.0)	0.045	78.8 ± 7.2 (67.8, 85.3)	83.6 ± 5.9 (75.0, 90.0)	0.099
TBR, %	4.6 ± 3.7 (1.0, 9.5)	6.3 ± 5.7 (2.0, 14.0)	0.218	4.3 ± 2.4 (1.6, 6.7)	4.8 ± 3.1 (2.0, 10.0)	0.193	5.9 ± 4.7 (1.7, 13.3)	2.6 ± 1.8 (1.0, 5.0)	0.081
TAR, %	23.8 ± 5.4 (19.0, 31.5)	20.0 ± 3.9 (16.0, 25.0)	0.415	26.6 ± 5.9 (18.8, 31.8)	16.6 ± 5.9 (8.0, 22.0)	0.045	15.3 ± 4.4 (6.0, 20.0)	13.8 ± 5.4 (6.0, 20.0)	0.534
GMI, mmol/mol	43.5 ± 2.4 (41.4, 47.1)	42.7 ± 0.2 (42.4, 42.8)	0.581	44.0 ± 1.4 (42.8, 45.7)	41.4 ± 1.9 (39.5, 44.2)	0.067	40.0 [39.5–40.9]	42.8 [41.9–42.8]	0.345
CV, %	33.8 ± 5.4 (26.6, 39.7)	32.1 ± 7.3 (26.6, 42.2)	0.725	29.7 [28.1–31.9]	28.4 [27.4–29.3]	0.080	29.5 ± 4.1 (25.0, 36.2)	26.1 ± 5.1 (21.0, 34.4)	0.007
HbA_1c_, %	5.6 ± 0.4 (5.0, 6.0)	5.8 ± 0.3 (5.3, 6.1)	0.310	5.5 ± 0.4 (5.0, 6.0)	5.7 ± 0.3 (5.1, 6.0)	0.205	6.0 ± 0.3 (5.5, 6.3)	6.0 ± 0.3 (5.6, 6.3)	0.749
mmol/mol	35.2 ± 4.8 (31.2, 42.1)	39.3 ± 3.1 (34.4, 43.2)		36.4 ± 4.2 (31.2, 42.1)	38.3 ± 3.5 (32.2, 42.1)		41.6 ± 3.5 (36.6, 45.4)	42.1 ± 3.0 (37.7, 45.4)	
Total insulin (IU)	40.3 ± 14.4 (29.4, 61.1)	42.0 ± 9.6 (33.6, 55.8)	0.614	47.9 ± 21.0 (34.3, 90.2)	43.7 ± 9.7 (33.6, 59.5)	0.494	69.8 ± 40.6 (41.2, 141.3)	66.8 ± 16.8 (52.3, 90.4)	0.822
Total insulin per body weight (IU/kg)	0.55 ± 0.17 (0.38, 0.76)	0.56 ± 0.10 (0.47, 0.65)	0.868	0.66 ± 0.21 (0.49, 1.1)	0.60 ± 0.11 (0.44, 0.72)	0.518	0.89 ± 0.36 (0.63, 1.52)	0.89 ± 0.18 (0.67, 1.14)	0.987
Total bolus insulin (IU)	22.0 ± 8.0 (15.5, 33.6)	23.4 ± 4.5 (17.1, 27.9)	0.792	27.7 ± 14.9 (13.7, 57.0)	31.3 ± 5.3 (24.5, 36.8)	0.516	29.4 [22.3–31.4]	44.2 [41.9–59.4]	0.500
Total bolus insulin per body weight (IU/kg)	0.30 ± 0.09 (0.20, 0.42)	0.31 ± 0.07 (0.24, 0.39)	0.837	0.37 ± 0.15 (0.24, 0.67)	0.44 ± 0.09 (0.32, 0.55)	0.430	0.49 [0.33–0.46]	0.65 [0.65–0.73]	0.138
Daily bolus insulin (%)	54.8 ± 5.4 (49.0, 62.0)	57.0 ± 12.7 (43.0, 72.0)	0.798	59.0 [56.0–61.0]	74.0 [73.0–75.0]	0.046	41.0 ± 10.3 (41.0, 68.0)	76.2 ± 3.8 (72.0, 80.0)	0.015
Total basal insulin (IU)	18.3 ± 7.0 (12.0, 27.3)	18.6 ± 9.1 (10.9, 31.8)	0.908	18.6 [16.6–20.6]	11.2 [9.1–11.9]	0.028	27.6 [27.1–28.8]	17.2 [48.0–55.0]	0.043
Total basal insulin per body weight (IU/kg)	0.25 ± 0.09 (0.17, 0.34)	0.24 ± 0.10 (0.13, 0.37)	0.856	0.28 ± 0.08 (0.20, 0.39)	0.17 ± 0.04 (0.12, 0.25)	0.005	0.42 [0.39–0.42]	0.19 [0.16–0.27]	0.043
Daily basal insulin (%)	45.3 ± 5.4 (38.0, 51.0)	43.0 ± 12.7 (28.0, 57.0)	0.798	41.0 [39.0–44.0]	26.0 [25.0–27.0]	0.046	48.4 ± 10.3 (32.0, 59.0)	23.8 ± 3.8 (20.0, 28.0)	0.015
Daily carbs (g)	177.0 ± 45.2 (120.0, 227.0)	166.8 ± 92.7 (98.0, 298.0)	0.815	204.5 ± 69.7 (144.0, 313.0)	221.0 ± 58.6 (124.0, 282.0)	0.726	211.8 ± 60.4 (143.0, 288.0)	235.0 ± 53.6 (152.0, 287.0)	0.648
Number of meals	8.7 ± 3.5 (5.1, 12.8)	5.5 ± 3.8) (2.5, 11.1)	0.058	9.1 ± 1.8 (6.5, 11.1)	9.4 ± 3.7 (3.9, 14.6)	0.849	9.9 ± 0.7 (9.1, 10.7)	9.6 ± 3.6 (3.9, 12.8)	0.839
Sensor time, %	79.8 ± 19.2 (55.6, 95.5)	70.8 ± 18.6 (48.0, 90.0)	0.531	81.0 ± 12.2 (62.2, 94.0)	83.0 ± 25.8 (37.0, 97.0)	0.879	90.2 ± 5.7 (82.9, 96.1)	94.2 ± 3.4 (90.0, 97.0)	0.112

SG—sensor glucose concentration, GMI—glucose management indicator, CV—coefficient of variation, HbA_1c_—glycated hemoglobin; TBR—time below range (glucose concentration < 3.5 mmol/L), TAR—time above range (glucose concentration > 7.8 mmol/L). Data are presented as means ± standard deviation for normally distributed variables or mean [interquartile range] for variables that deviated from the normal distribution.

**Table 3:** Association of the number of meals/daily carbs with glycemic parameters for the second pregnancy (on HCLs), described using the Pearson r correlation coefficient.

	Number of Meals			Daily Carbs		
	First Trimester	Second Trimester	Third Trimester	First Trimester	Second Trimester	Third Trimester
	r *p*	r *p*	r *p*	r *p*	r *p*	r *p*
Daily carbohydrates, g	0.936 0.006	0.855 0.030	0.752 0.143	/	/	/
Mean SG, mmol/L	−0.427 0.399	−0.225 0.668	−0.632 0.253	−0.223 0.672	0.185 0.726	−0.507 0.384
TIR, %	0.550 0.259	0.628 0.182	0.987 0.002	0.631 0.179	0.508 0.303	0.727 0.164
TBR, %	−0.443 0.379	−0.559 0.249	−0.532 0.357	−0.587 0.221	−0.805 0.053	−0.411 0.492
TAR, %	−0.623 0.187	−0.509 0.303	−0.891 0.042	−0.618 0.191	−0.251 0.631	−0.650 0.235
GMI, mmol/mol	−0.427 0.399	−0.225 0.668	−0.632 0.253	−0.223 0.672	0.185 0.726	−0.507 0.384
CV, %	−0.542 0.267	−0.776 0.070	−0.914 0.030	−0.635 0.176	−0.766 0.076	−0.668 0.218
HbA_1c_, %	0.482 0.333	0.122 0.818	0.043 0.945	0.618 0.191	0.373 0.466	0.645 0.251
Total insulin (IU)	−0.702 0.120	−0.813 0.049	−0.970 0.006	−0.501 0.312	−0.671 0.145	−0.678 0.209
Total bolus insulin per body weight (IU/kg)	−0.614 0.195	−0.115 0.828	−0.527 0.361	−0.484 0.331	−0.245 0.639	−0.391 0.515
Total bolus insulin (IU)	0.061 0.909	−0.680 0.137	−0.932 0.021	0.382 0.455	−0.451 0.369	−0.681 0.205
Total bolus insulin per body weight (IU/kg)	0.620 0.190	0.189 0.720	−0.349 0.565	0.664 0.150	0.088 0.868	−0.301 0.622
Daily bolus insulin, %	0.939 0.005	0.714 0.111	0.734 0.158	0.907 0.013	0.790 0.061	0.394 0.632
Total basal insulin (IU)	−0.834 0.039	−0.818 0.047	−0.979 0.004	−0.736 0.095	−0.785 0.064	−0.625 0.260
Total basal per body weight (IU/kg)	−0.914 0.011	−0.618 0.191	−0.872 0.054	−0.837 0.038	−0.785 0.095	−0.535 0.353
Daily basal insulin, %	−0.939 0.005	−0.714 0.111	−0.734 0.158	−0.907 0.013	−0.790 0.061	−0.294 0.632
Autocorrection bolus, %	0.658 0.543	−0.304 0.558	−0.957 0.011	0.996 0.054	0.039 0.942	−0.802 0.103
Sensor time, %	0.549 0.259	0.760 0.079	0.847 0.070	0.736 0.096	0.825 0.043	0.591 0.294

SG—sensor glucose concentration, GMI—glucose management indicator, CV—coefficient of variation, HbA_1c_—glycated hemoglobin; TBR—time below range (glucose concentration < 3.5 mmol/L), TAR—time above range (glucose concentration > 7.8 mmol/L).

**Table 4:** Summary of findings.

When Using the HCLs during Pregnancy
-women spent more time in TIR (3.5–7.8 mmol/L or 63–140 mg/dL) than when using SAPs.
-women spent less time in TAR (above 7.8 mmol/L or 140 mg/dL) than when using SAPs.
-had lower glucose variability than when using SAPs.
-higher meal frequency was associated with higher TIR.
-higher meal frequency was associated with lower TAR.
-higher meal frequency was associated with lower glycemic variability.

TIR—time in range, TBR—time below range (glucose concentration < 3.5 mmol/L), TAR—time above range (glucose concentration > 7.8 mmol/L).

## Data Availability

The data presented in this study are available on request from the corresponding author. The data are not publicly available due to privacy.

## Supplementary Materials

The following supporting information can be downloaded at: https://www.mdpi.com/article/10.3390/metabo12111137/s1, Table S1: Associations between the number of meals and daily carbs with glycemic parameters for the first pregnancy (on SAP), described by the Pearson r correlation coefficient, Table S2: Self-reported physical activity during the first and the second pregnancy.
