# Supplementary material for: Carbohydrate Intake and Closed-Loop Insulin Delivery System during Two Subsequent Pregnancies in Type 1 Diabetes
Source: Metabolites. 2022 Nov 18;12(11):1137. doi: 10.3390/metabo12111137 (PMC9699352; doi:10.3390/metabo12111137)
Supplement: Supplementary file 1 [file metabolites-12-01137-s001.zip › metabolites-1996969-supplementary.pdf]

Supplementary Table S1. Associations between number of meals and daily carbs with glycemic parameters for the first pregnancy (on SAP), described by the Pearson r correlation coefficient.

	Number of meals			Daily carbs		
	**First trimester**	**Second trimester**	**Third trimester**	**First trimester**	**Second trimester**	**Third trimester**
	r<br>p	r<br>p	r<br>p	r<br>p	r<br>p	r<br>p
Daily carbohydrates, g	0.527<br>0.473	0.351<br>0.495	0.230<br>0.661	/	/	/
Mean SG, mmol/l	-0.149<br>0.851	-0.238<br>0.700	0.079<br>0.882	-0.800<br>0.200	0.191<br>0.758	-0.187<br>0.722
TIR, %	0.925<br>0.075	0.405<br>0.499	0.260<br>0.619	**0.807**<br>**0.193**	-0.042<br>0.946	-0.245<br>0.640
TBR, %	-0.718<br>0.282	0.578<br>0.307	-0.181<br>0.732	0.015<br>0.985	0.592<br>0.293	0.381<br>0.457
TAR, %	-0.579<br>0.421	-0.687<br>0.200	-0.227<br>0.666	-0.948<br>0.052	-0.188<br>0.762	-0.010<br>0.985
GMI, mmol/mol	-0.515<br>0.584	-0.238<br>0.700	0.079<br>0.882	-0.013<br>0.987	0.191<br>0.758	-0.187<br>0.722
CV, %	-0.557<br>0.443	0.101<br>0.872	-0.692<br>0.128	0.344<br>0.656	0.673<br>0.213	0.035<br>0.948
HbA1c, %	-0.323<br>0.677	-0.624<br>0.186	0.577<br>0.230	0.052<br>0.948	-0.354<br>0.491	0.116<br>0.827
Total insulin (IU)	-0.856<br>0.144	-0.159<br>0.765	-0.206<br>0.695	-0.014<br>0.986	0.710<br>0.114	0.709<br>0.115
Total insulin per body weight (IU/kg)	**-0.975**<br>**0.025**	-0.020<br>0.970	-0.044<br>0.934	-0.329<br>0.671	0.665<br>0.149	0.654<br>0.159
Total bolus insulin (IU)	-0.786<br>0.214	-0.184<br>0.727	-0.237<br>0.650	0.055<br>0.945	0.764<br>0.077	0.694<br>0.159
Total bolus insulin per body weight (IU/kg)	-0.926<br>0.074	-0.117<br>0.826	-0.166<br>0.753	-0.237<br>0.763	0.779<br>0.068	0.666<br>0.149
Daily bolus insulin, %	0.263<br>0.737	-0.320<br>0.536	-0.465<br>0.352	0.233<br>0.767	0.629<br>0.181	0.640<br>0.171
Total basal insulin (IU)	-0.875<br>0.065	-0.087<br>0.870	-0.086<br>0.871	-0.092<br>0.908	0.521<br>0.290	0.756<br>0.082
Total basal insulin per body weight (IU/kg)	-0.935<br>0.065	0.174<br>0.741	0.436<br>0.387	-0.391<br>0.609	0.231<br>0.660	0.446<br>0.375
Daily basal insulin, %	-0.263<br>0.737	0.320<br>0.536	0.465<br>0.352	-0.233<br>0.767	-0.629<br>0.181	-0.640<br>0.171
Sensor time, %	0.233<br>0.767	0.777<br>0.122	0.450<br>0.370	0.948<br>0.052	0.434<br>0.466	0.252<br>0.630

SG-sensor glucose concentration, GMI-glucose management indicator, CV-coefficient of variation, HbA_{1c}-glycated hemoglobin; TBR–time below range (glucose concentration <3.5 mmol/l), TAR – time above range (glucose concentration >7.8 mmol/l)

Supplementary Table S2. Self-reported physical activity during the first and the second pregnancy

	First pregnancy	Second pregnancy	p value
Frequency of PA	2.5±1.4	3.3±1.5	0.289
Time of PA	2.0±0.6	1.8±0.8	0.611
Intensity of PA	2±0.0	2.0±0.7	1.000

PA-physical activity

Frequency of PA: from 1- twice per week or less to 5-multiple times per day.

Time of PA: 1-30 minutes or less, 2-between 30 and 60 minutes, 3-60 minutes or more.

Intensity of PA: 1-low, 2-moderate, 3-high
